# Strategies to Screen and Evaluate Brain Targeting Antibodies Using an iPSC-Derived Blood–Brain Barrier Model

**DOI:** 10.3390/antib14040102

**Published:** 2025-11-26

**Authors:** Eun Seo Choi, Sophia Sahota, Emily Burnham, Yunfeng Ding, Eric V. Shusta

**Affiliations:** 1 Department of Chemical and Biological Engineering, University of Wisconsin-Madison, Madison, WI 53706, USA; 2 Department of Neurological Surgery, University of Wisconsin-Madison, Madison, WI 53706, USA

**Keywords:** BBB transport, antibody engineering, CNS therapeutic development, directed evolution, high-throughput screening, BBB transcytosis, histidine mutagenesis, CDR

## Abstract

Background: Antibodies that cross the blood–brain barrier (BBB) by targeting receptor-mediated transport (RMT) systems can allow efficient drug delivery to the central nervous system (CNS). In order to improve brain uptake of antibodies, their binding properties have been engineered, but it is not always clear what antibody properties dictate BBB transport efficiency. In this study, we therefore developed and employed an in vitro phenotypic screen and a quantitative transcytosis assay in an attempt to identify improved variants of a previously identified BBB transcytosing antibody known as 46.1. Methods: First, a random mutagenic 46.1 antibody phage display library was screened for improved transcytosis through a human induced pluripotent stem cell (iPSC)-derived BBB model. These screens yielded antibody variants that enriched over multiple screening rounds; however, when produced as soluble antibodies, the variants did not display improved in vitro transcytosis over the wild-type (WT) 46.1 antibody. As a second strategy, we performed a targeted histidine point mutation of a solvent-exposed residue in each complementarity-determining region (CDR) and evaluated the in vitro transcytosis capacity of the variants. Results and Conclusions: In this way, we identified a 46.1 variant, R162H, with modestly improved in vitro transcytosis properties. These results show that the iPSC-derived BBB screening insights and evaluation strategies presented here could facilitate the engineering and optimization of lead antibodies for CNS delivery.

## 1. Introduction

The blood–brain barrier (BBB) plays a crucial role in maintaining brain homeostasis but presents a stringent barrier leading to poor penetration of biotherapeutics targeting central nervous system (CNS) diseases [1,2]. Resultant limited bioavailability of systemically administrated therapeutics necessitates elevated dosing which can trigger adverse responses and lead to prohibitive costs [3,4]. In an attempt to address these issues, there is ongoing development of antibodies that can target and co-opt receptor-mediated transcytosis (RMT) transporters at the BBB for drug delivery to the CNS. In this way, therapeutics conjugated to an RMT-targeted antibody can be delivered to the brain after peripheral administration. Examples of such brain shuttles currently in middle to late stage clinical trials include transferrin receptor (TfR) targeting antibodies such as Roche’s Trotinemab (NCT07169578) for the treatment of Alzheimer’s disease [5,6,7], Denali’s DNL310 (NCT05371613, NCT04251026, NCT06075537) [8,9,10] for the treatment of Mucopolysaccharidosis Type II (MPS-II), and JCR Pharmaceutical’s Pabinafusp alfa (JR-141) (NCT04573023, NCT05594992), which has been approved for patient use in Japan for the treatment of MPS-II [11,12].

While the conventional BBB RMT receptors such as the TfR and the insulin receptor (IR) show promise as brain delivery targets [5,6,8,9,13,14,15,16,17,18,19], the search for alternative RMT targets that offer additional benefits such as brain specificity and regiospecificity is also ongoing. To this end, various screening approaches have been used to identify alternative antibody-RMT pairs [20,21,22,23,24]. One BBB targeting antibody with direct relevance to this study, known as 46.1, was identified by mining a phage-display human antibody library against a human induced pluripotent stem cell (iPSC)-derived brain microvascular endothelial (BMEC) cell-like BBB model [24]. The 46.1 antibody binds, internalizes, and transcytoses across the iPSC-derived BBB model, and binds to the human and mouse BBB in brain tissue sections [24]. In addition, intravenous injection of 46.1 into mice demonstrates a significant brain accumulation [24], and when 46.1 is conjugated to neurotensin, the fusion exhibits a pharmacologic effect, demonstrating full trans-BBB delivery [25]. Although the BBB receptor targeted by clone 46.1 is currently unknown, it does not bind the commonly employed TfR or IR targets [24], offering the opportunity to further develop delivery vehicles against an alternative RMT target.

Although the 46.1 antibody accumulates in the CNS, it was selected from a nonimmune human antibody library and therefore has not been optimized for BBB delivery. Motivated by its potential, we sought to engineer 46.1 to improve its transcytosis properties. A multitude of factors, including the targeted receptor, the targeting antibody characteristics (affinity, avidity, valency, Fc region), and the dosage can influence the efficacy of RMT targeting antibodies [2]. While the optimization of affinity and valency has resulted in improved RMT-targeted antibodies, the rules governing the relationships between affinity, valency, and BBB transport efficiency can be unpredictable and vary for each antibody and RMT system [4,14,15,17,18,19,26,27,28,29,30,31,32,33,34]. To navigate the complexity of the RMT process, we implemented two complementary strategies. First, we developed a directed evolution pipeline which incorporates high-throughput transcytosis screens of a mutagenic phage-displayed 46.1 antibody library using the iPSC-derived BBB model. While phenotypic screens on several BBB models have been used to identify lead antibody candidates having the ability to transport across the BBB [20,21,23,24,25,35,36,37,38], using these screening systems for optimization of the lead antibodies has, to our knowledge, not yet been reported. Second, we explored a more targeted approach by introducing histidine substitutions into the most solvent-exposed residues within individual complementarity-determining regions (CDRs). CDRs play major roles in contacting the antigen and have therefore been subject to engineering to improve the binding affinity and specificity of antibodies [39,40,41,42,43,44,45,46]. In addition, studies have shown that tuning the pH sensitivity of the antibody-receptor binding interactions via histidine substitutions can improve intracellular trafficking capabilities across the BBB [44,47,48]. Using these antibody engineering strategies along with an in vitro transcytosis assay capable of detecting transcytosed proteins in the picomolar concentration range, we identified a variant that has improved in vitro transcytosis properties compared to the wild-type (WT) 46.1 antibody.

## 2. Materials and Methods

### 2.1. Library Construction

Random mutagenesis of single-chain variable fragment (scFv) 46.1 was performed by using nucleoside analog derivatives 8-oxo-dGTP and dPTP along with natural dNTPs, using Taq polymerase to incorporate into DNA [33]. PCR reactions were set up with High Fidelity Platinum Taq (Invitrogen, 10966018, Carlsbad, CA, USA), 50 mM MgCl_2_, fd-tet 46.1 plasmid as the template DNA [24,49,50], varying amounts of 8-oxo-dGTP (Tri-Link Biotech N-2034) and dPTP (Tri-Link Biotech N-2037, San Diego, CA, USA), and 10 mM of forward (5′-GACGTGTACTGCAGGAGTCGGGGGGAGGCTTAG-3′) and reverse (5′-AATTAATATGCGGCCGCACCTAGGACGGTCAGCTTGG-3′) primers flanking the 46.1 insert, containing PstI and NotI restriction sites, respectively. 8-oxo-dGTP produces A-to-C and G-to-T transversion mutations, while dPTP produces A-to-G and G-to-A transition mutations [51]. To preserve functionality and efficiently sample the sequence space [52], the number of PCR cycles and nucleoside analog concentrations were optimized to obtain one or two non-silent mutations of the total 243 residues of scFv 46.1. After sequencing 10–20 random colonies for each library, a combination of 15 PCR cycles and 2 µM of both 8-oxo-dGTP and dPTP was identified to give an average of 0.73 residues of non-silent mutations. Then, the mutagenized 46.1 sequence was cloned into the fd-tet vector [24,49,50], which can be expressed as a filamentous bacteriophage fd and has a tetracycline resistance, using the restriction sites PstI (NEB R3140S, Ipswich, MA, USA) and NotI (NEB R3189S). QuickCIP (NEB M0525S) was used to dephosphorylate the plasmid backbone during restriction digestion, and T4 DNA ligase (NEB M0202S) was used to ligate the plasmid and backbone at a 3:1 insert/vector ratio. Then, the ligation mix was electroporated into DH10B (NEB C3020K) and outgrown in the NEB10-beta/Stable Outgrowth medium supplied with the DH10B cells for an hour to generate the plasmid DNA library. A plating assay was performed to determine the library size. Briefly, serial dilutions of the outgrowth mixture were plated, the plates were incubated at 37 °C overnight, the colonies were counted, and the library size was calculated according to the dilution factors.

### 2.2. Phage Display

To prepare the phage for screening, TG1 *E. coli* (Agilent 200123, Santa Clara, CA, USA) was electroporated with either the 46.1 random mutagenesis DNA library or the negative control ABN (anti-botulinum neurotoxin scFv) in the fd-tet plasmid backbone [24,49,50] and was grown overnight at 37 °C in 150 mL of 2xYT/tet medium. The next day, the bacterial mixture was spun down, and the phage-containing supernatant was precipitated with 1/5 volume of 20% *w*/*v* PEG8000 in 2.5 M NaCl on ice for an hour. Then, the precipitated mixture was spun down and the pellet containing the fd phage was resuspended in PBS at 1/10 volume of the culture. For phage quantification, 10-fold serial dilutions of phage were made, from 10× to 10^7^× on a 96-well plate, and the 10^7^× diluted phage was incubated with log-phase TG1 *E. coli* in a 1:1 volumetric ratio for 30 min at 37 °C. Then, 10-fold serial dilutions of the mixture of 10^7^× diluted phage and TG1 were made, from 2 × 10^7^ to 2 × 10^13^×, and 10 µL of each dilution mixture were spotted on a dried 2xYT/tet agar plate. The agar plate was incubated at 37 °C overnight, colonies were counted the next day for each dilution, and the number of phages was calculated.

### 2.3. Next Generation Sequencing (NGS)

The insert region encoding the scFv 46.1 or mutants were amplified using the Q5 High-Fidelity DNA Polymerase (NEB M0492) and the following forward and reverse primers: 5′-TTTTTGGAGATTTTCAACGTGA-3′ and 5′-GAATTTTCTGTATGAGGTTTTGCTAAA-3′. The PCR product was purified using a Zymo DNA Clean & Concentrator kit (Zymo Research D4004, Irvine, CA, USA) and submitted to Genewiz (South Plainfield, NJ, USA) for PacBio sequencing. PacBio SMRTbell libraries were prepared and sequenced on the PacBio Sequel IIe platform. Using the project deliverables in the FASTQ format, the DNA sequences of the region encoding scFv 46.1 and its mutants were translated into protein sequences, and the mutation frequency at each amino acid position and the frequency of occurrence of each clone were evaluated. NGS was performed for the initial library.

### 2.4. Phage Displayed Antibody Library Transcytosis Screen on iPSC-BMEC-like Cells

BMEC-like cells were differentiated using the UM and retinoic acid induction method with the IMR90-C4 iPSC line as previously described [53,54]. Briefly, the IMR90-4 cells were expanded on Matrigel-coated plates in mTeSR1 medium (Stem Cell Technologies 85850, Vancouver, BC, Canada) for 3 days. Differentiation was initiated by culturing colonies in unconditioned medium (DMEM/F-12 (Gibco 11330-057, Grand Island, NY, USA) containing 20% Knockout Serum Replacer (Gibco 10828-028), 1× MEM nonessential amino acids (Gibco 11140-050), 1 mM l-glutamine (Gibco 35050-061), and 0.1 mM β-mercaptoethanol (Sigma M3148, Burlington, MA, UA) for 5 days [53,54]. On day 6, the medium was changed into endothelial cell (EC) medium (human Endothelial Serum-Free Medium (Gibco 11111-044), 1× B27 (Gibco 17504044)) supplemented with 10 µM retinoic acid (RA) and 20 ng/mL human fibroblast growth factor 2 (FGF2) [53,54]. On day 8, BMECs were subcultured on collagen IV/fibronectin coated 1 µm pore size transwells (Falcon 353103, Corning, NY, USA) in EC medium supplemented with RA and FGF2 [53,54]. On day 9, the medium was changed into EC medium. Trans-endothelial electrical resistance (TEER) was measured on days 9 and 10 [53,54]. We performed the transcytosis screens on the day at which the BMEC-like cells exhibit maximum TEER (day 10 after initiating differentiation) in order to maximize reproducibility in barrier properties amongst different differentiations. The transcytosis screens were performed with phage-displayed scFv 46.1 mutant library and the negative control scFv ABN to identify clones which exhibit the highest accumulation in the basolateral chamber. In more detail, the cell media were changed into transport buffer (HBSS (Gibco 14025092) supplemented with 5 mM magnesium chloride and 10 mM HEPES (Gibco 15630080)). Cells were incubated in 37 °C at 5% CO_2_ for the TEER to stabilize for two hours. Then, 10^11^ CFU of phage library, or Round I output pool in the case of the 90 min screen, along with 10 µM sodium fluorescein (NaF), were dosed on the apical chamber of the transwell insert with iPSC-BMEC-like cells with TEER values exceeding 1000 Ω cm^2^. The transwell inserts were incubated at 37 °C and 5% CO_2_ for various time points: 60 min, 90 min, and 120 min. Subsequently, log phase TG1 *E. coli* was infected with the entire basolateral chamber content, and the bacteria harboring the phage were plated on 2xYT/tet agar to count the number of CFUs of phage that crossed the iPSC-BBB model. Alongside, the NaF content of the basolateral chamber was read on a plate reader to assess the barrier integrity. NaF has a size sufficiently small to allow paracellular diffusion through the BBB model and therefore serves as a measure of BBB permeability and ensures that barrier disruption is not leading to increased phage passage [55]. The colonies formed by the phage displaying the scFv that passed through the BBB model were picked and sequenced. Alongside, all the colonies from the 90 min Round I screen output were scraped to make a pooled library, and the phage transcytosis screen was repeated on the iPSC-BMECs for the 90 min Round II screen.

### 2.5. Computational Modeling of WT 46.1 and Its Variants

The primary sequence of the WT scFv 46.1 was input into the I-TASSER server for homology modeling [56,57,58]. With the I-TASSER solvent accessibility predictions, one residue per CDR, with the highest predicted solvent accessibility, was mutated into histidine.

### 2.6. Protein Production

For the production of variants in scFv-Fc formats, the insert regions of the lead candidates in the fd-tet backbone were PCR amplified using the forward (5′- GATACTTAAGCTAGCCAGGTGCAGCTGCAGGAGT-3′) and reverse (5′- TCATTAGAACCGGTACCTAGGACGGTCAGCTTGGTC-3′) primers containing the NheI and AgeI restriction sites, respectively. The insert was cloned into the place of WT 46.1 insert in the pIRES-46.1-rabbitFc plasmid [24] using the NheI (NEB R3131S) and AgeI (NEB R3552S) restriction enzymes. For the histidine point mutants, gene blocks for each variant were codon optimized for *Cricetulus griseus* using the Integrated DNA Technologies (IDT, Coralville, IA, USA) codon optimization tool. gBlocks (IDT) for each variant were designed so they contained the NheI and AgeI restriction sites in the 5′- and 3′-ends of the variant sequence. These gBlocks were cloned into the place of the scFv 46.1 insert in the pIRES-scFv 46.1-rabbitFc plasmid using the NheI and AgeI restriction sites. For the production of scFv-Fc-nanoluciferase (nLuc) soluble proteins, an scFv-Fc-nLuc plasmid construct was designed so the nLuc portion is linked to the C-terminus of the rabbit Fc region via a long linker ((G_3_S)_2_). A gene block consisting of part of the rabbit Fc region containing the BbvCI (NEB R0601S) restriction site and a nLuc portion flanked by a NotI restriction site (NEB R3189S) was subcloned into the pIRES-scFv-rabbitFc plasmids using the respective restriction sites. The plasmids were ligated with T4 ligase, transformed into NEB 5-alpha Competent *E. coli* (NEB C2987H), and plated on LB agar plates containing carbenicillin.

Protein expression was performed using the ExpiCHO^TM^ Expression System (Gibco A29133). Briefly, the ExpiCHO cells were cultured to reach 6M viable cells per mL and transfected with about 40 µg of plasmid DNA, ExpiFectamine™ CHO Reagent, and OptiPRO™ medium. The next day, ExpiFectamine™ CHO Enhancer and ExpiCHO™ Feed were added. Five to seven days after the transfection, the transfected cells were collected and centrifuged at 3200 RCF at 4 °C. The supernatant was combined with 5× binding buffer (0.5 M Sodium Phosphate, 0.25 M Sodium chloride, pH 7.4) and protein A/G agarose resin (Pierce 20423, Rockford, IL, USA), and incubated shaking overnight at 4 °C. The next day, column purification of the Fc proteins was performed. Briefly, the columns were equilibrated with 1x binding buffer, and the supernatant containing the scFv-Fc-bound A/G resin was added. The columns were washed with 1x binding buffer and 1x wash buffer (0.1 M sodium phosphate, 0.3 M sodium chloride, pH 7.4) and eluted with elution buffer (0.1 M citric acid, pH 3) plus one-tenth volume of the neutralization buffer (1 M Tris pH 9). Proteins were dialyzed with PBS in a 10k Molecular Weight Cut-Off (MWCO) dialysis cassette (Life Technologies A52971, Carlsbad, CA, USA) and run on SDS-PAGE and stained with Coomassie for size validation.

### 2.7. Membrane Binding and Endocytosis Assay

The iPSC-derived BMEC-like cells were subcultured onto LabTekII slides (Nunc #154917, Roskilde, Denmark) on day 8 of differentiation. The BMEC-like cells were blocked with 10% goat serum in PBS (PBSG) for 30 min on ice. 5 µg/mL of scFv-Fc proteins were added to the BMEC-like cells, incubated for 30 min on ice for binding, and 30 min at 37 °C for internalization. Then, the cells were washed with cold PBS and incubated with goat anti-rabbit AlexaFluor555-conjugated secondary antibody (Invitrogen A21428), 1:1000 in PBSG, for 30 min on ice to label the cell surface-bound scFv-Fc fraction. The cells were fixed and permeabilized with 4% PFA and 0.2% Triton-X, respectively. Then, the cells were incubated with goat anti-rabbit AlexaFluor488-conjugated secondary antibody (Invitrogen A11008), 1:1000 in PBSG, at room temperature to label the internalized scFv fraction. Finally, the cells were washed and mounted with ProLong Gold anti-fade reagent with DAPI (Invitrogen P36935). Images were taken using a Nikon Ti2-E microscope and analyzed with ImageJ (Version 2.3.0).

### 2.8. Luciferase-Based Transcytosis Assay

Protein concentrations were quantified using the Qubit system. Proteins were run on SDS-PAGE and stained with Coomassie and Nano-Glo^®^ In-Gel Detection System (Promega N3020, Madison, WI, USA) for size validation and nLuc functional validation, respectively. For the transcytosis assay, the iPSC-BMEC-like cells were subcultured on transwells with 1 µm pore size (Falcon 087718) on day 8 of differentiation. On day 10, TEER of the cells was measured as described above for the phage transcytosis screens. Then, the cell media were changed into transport buffer. Cells were incubated in 37 °C at 5% CO_2_ for the TEER to stabilize for two hours. Then, to the cells with TEER values exceeding 1000 Ω cm^2^, 153 nM of the scFv-Fc-nLuc proteins were dosed on the apical chamber. The transwells undergoing the transport assay were incubated in 37 °C at 5% CO_2_ shaking at 30 rpm for four hours. After the incubation, the media from the basolateral chamber containing the transcytosed scFv-Fc-nLuc were collected for analysis. The Nano-Glo^®^ Luciferase Assay System (Promega N1110) was used to generate the luminescence signal with the nLuc enzyme upon addition of the furimazine substrate, and the luminescence values were read on a Tecan Infinite 200 PRO plate reader. Calibration curves were used to measure the concentrations.

## 3. Results

### 3.1. Antibody Library Creation, Screening, and Lead Candidate Selection

We hypothesized that mutations in the 46.1 antibody can alter its binding affinity, receptor engagement, or trafficking in a way that benefits RMT across the BBB. Given that the target receptor of the 46.1 antibody is currently unknown, limiting rational design strategies, we employed a random mutagenesis approach to explore the transcytosis fitness landscape. We constructed a randomly mutagenized library of the 46.1 antibody in a phage-displayed single-chain antibody scFv format, introducing an average of 0.73 coding mutations per scFv mutant, resulting in a library size of 2.5 × 10^5^. The resulting library had mutations evenly distributed throughout the length of the scFv into many of the 20 possible amino acid residue combinations, with mutational frequencies ranging from 0.2 to 0.6%, except for P192L, which appeared in an anomalously high percentage of the clones (Supplementary Figure S1a). Mutations were spread out across the framework and CDRs, except at the ends of the scFv sequence where mutation rates were lowest, owing to the PCR-based mutagenic strategy (Supplementary Figure S1a,b).

**Figure 1:**
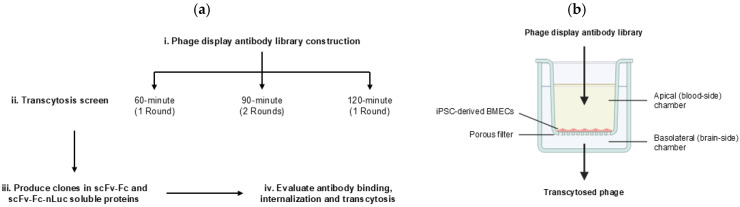
Screen workflow. (**a**) i. Phage display mutagenic 46.1 library was constructed. ii. Transcytosis screens were performed on iPSC-derived BMEC-like cells cultured on transwell inserts for three different screening times: 60 min, 90 min, and 120 min. For the 90 min screen, the Round I outputs were pooled and screened again for a second round. iii. Lead candidates were produced in scFv-Fc and scFv-Fc-nLuc formats and were iv. Evaluated for binding, internalization, and transcytosis across the iPSC-derived BMEC-like cells. (**b**) Schematic of transwell set up of the iPSC-derived BBB model. Phage library was added to the apical chamber and transcytosed phage were recovered from the basolateral chamber.

**Figure 2:**
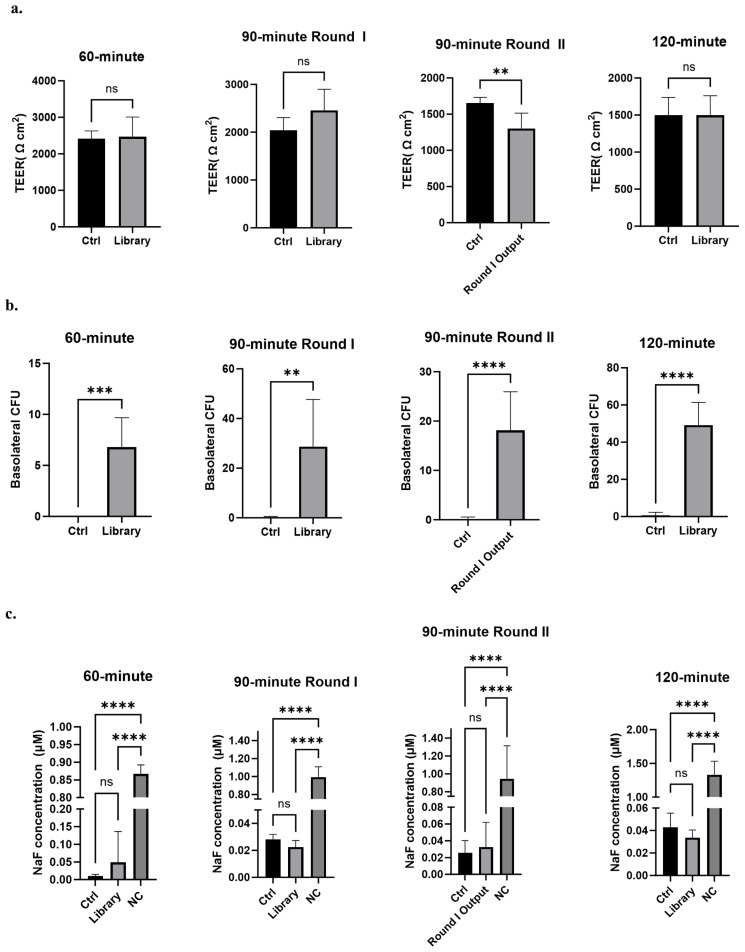
Library screening outputs. (**a**) TEER of iPSC-BMEC-like cell coated transwells used for each phage transcytosis screen. Analyzed with unpaired Student’s *t*-test. (**b**) Phage recovered in the basolateral chamber expressed as colony forming units (CFU). Analyzed with unpaired Student’s *t*-test. For all panels, n = 5 transwells for 60 min screen; n = 6 for 90 min Round I and 120 min screens; n = 6 for ABN and n = 10 for library for 90 min Round II. (**c**) Sodium fluorescein (NaF) accumulation in the basolateral chamber of transwells for each phage transcytosis screen at the end of the assay. Analyzed with one-way ANOVA followed by Tukey’s multiple comparisons test. Ctrl is anti-botulinum (ABN) scFv-displaying negative control phage; NC is a control transwell with no cells present. ** *p* ≤ 0.01, *** *p* ≤ 0.001, **** *p* ≤ 0.0001, ns: not significant.

The phage display 46.1 variant library was screened for the phenotype of improved transcytosis across the iPSC-derived BBB model (Figure 1). The iPSC-derived BMEC-like cells used for screening were differentiated in the same way as they were for the initial identification of the 46.1 antibody [24,53,54]. As previously described [24], it is crucial for the cell monolayer to be very tight, as measured by TEER values above 1000 Ω cm^2^, to prevent passive phage leakage from the apical to the basolateral chamber which would mask the identification of improved mutants. To this end, the iPSC-derived BMEC-like cell monolayers expressed key tight junction proteins occludin and claudin-5 as expected [53,54] (Supplementary Figure S2), and the iPSC-derived BMECs employed for the screens all had TEER values exceeding 1000 Ω cm^2^ (Figure 2a). To distinguish improved variants from WT 46.1, a screening pressure of decreased transcytosis time was applied for multiple rounds (Figure 1 and Figure 2). For the screens, we used three transcytosis times of 60, 90, and 120 min that were shorter than the 180 min used for the original identification of 46.1 [24]. We screened the library on at least five parallel iPSC-BMEC-like cell coated transwells for each transcytosis time and round. Phage (10^11^ CFU) were added to the apical chamber and transcytosing phage were recovered from the basolateral chamber at the prescribed screening times. The number of transcytosed phage recovered from the basolateral chamber was significantly higher for the library compared to the negative control scFv-displaying phage for all screening times (Figure 2b). Longer transcytosis incubation times generally resulted in higher numbers of phage recovered in the basolateral chamber, indicating the impact of the transcytosis time screening pressure. Importantly, the passive diffusion of a hydrophilic tracer, fluorescein, was measured in each transwell over the course of the screen. There were no differences in the accumulation of fluorescein in the basolateral chamber between the negative control scFv-displaying phage and the library, indicating that the phage library itself was not disrupting the iPSC-BMEC monolayer, resulting in the observed increased phage passage (Figure 2c). While the 60 min screen yielded an average of about 7 transcytosed phage per transwell (34 Total phage), the 90 min screen yielded about 29 transcytosed phage per transwell (172 Total phage), and the 120 min screen yielded about 50 transcytosed phage per transwell (295 Total phage) (Figure 2b). Given the few colonies in the 60 min screen and the lower stringency screening pressure of the 120 min screen, we additionally screened the pooled transcytosing phage from Round I of the 90 min screen for a second round. The Round II 90 min screen yielded about 18 colonies per transwell (181 total phage). Sequencing of the transcytosed pools and comparison to the library inputs allowed for the determination of antibody clones that were both relatively low frequency in the library input and enriched through the transcytosis screens (Table 1 and Supplementary Figure S3). Interestingly, while several clones enriched substantially from the library through rounds I and II of the 90 min screens, the WT enriched only slightly (Table 1), suggesting that those clones that do enrich in successive 90 min screens are performing differently than phage that display WT 46.1. Interestingly, the most highly enriched candidates in the 90 min screens possessed mutations in the framework region rather than the CDRs, despite there being variants with CDR mutations in the transcytosing pools (Supplementary Figure S3). From this analysis, we selected six lead candidates (RM1-RM6) that either had the highest enrichment in the two successive rounds of 90 min screens (RM 1, 2, 3, and 6) or had reasonably high enrichment and also appeared in the 60 min (RM 4 and 5) and/or 120 min screen outputs (RM 5) (Table 1, Supplementary Figure S3).

### 3.2. Evaluation of Binding, Internalization, and Transcytosis of Antibody Variants from the Screening Outputs in the iPSC-Derived BBB Model

**Figure 3:**
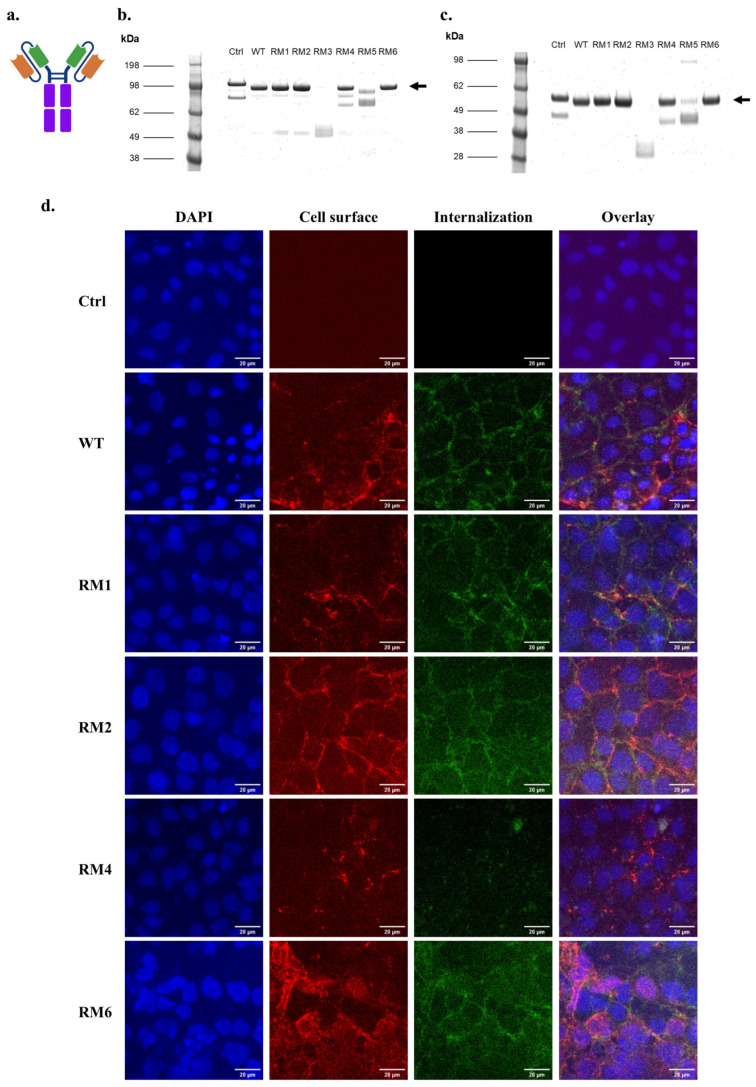
scFv-Fc binding and internalization into iPSC-BMECs for high-throughput screen outputs. (**a**) Schematic of the scFv-Fc structure. Orange: Variable heavy chain (VH), Green: Variable light chain (VL), Purple: Fc region. (**b**) Coomassie-stained non-reducing SDS-PAGE of purified, soluble scFv-Fc. Arrow marks the size of full-length, dimeric scFv-Fc. (**c**) Coomassie-stained reducing SDS-PAGE of purified, soluble scFv-Fc. Arrow marks the size of one arm of the scFv-Fc. (**d**) Cell surface association and internalization of soluble scFv-Fc protein into iPSC-BMECs after 30 min incubation at 37 °C. Scale bars are 20 µm. Ctrl is 4420-Fc negative control.

We next produced and purified soluble proteins in scFv-Fc formats for the lead candidates RM1-RM6 (Figure 3a). SDS-PAGE analysis followed by Coomassie staining indicated that full-length scFv-Fc could be produced for the WT 46.1 and the variants RM1, RM2, RM4, and RM6, producing a dominant full-length band at the expected size of about 100 kDa (Figure 3b,c). A negative control scFv-Fc fusion was also produced for 4420, an scFv which binds to fluorescein and therefore does not cross the BBB. The 4420-Fc and RM4-Fc also had some breakdown products at a lower molecular weight of about 80 kDa (Figure 3b). RM3 and RM5 were not produced well as full-length proteins and were not evaluated further (Figure 3b,c). To confirm that the variants maintained the capability to bind and internalize into iPSC-derived BMEC-like cells and traffic to the cell junctions as was previously demonstrated for WT 46.1 [24], the scFv-Fcs were pulsed onto iPSC-derived BMEC-like cells and allowed to bind and traffic for 30 min each. Each of the variants was able to bind and internalize into the iPSC-derived BMEC-like cells and traffic to the cell junctions, although qualitatively, RM4 demonstrated less total cellular association (Figure 3d).

**Figure 4:**
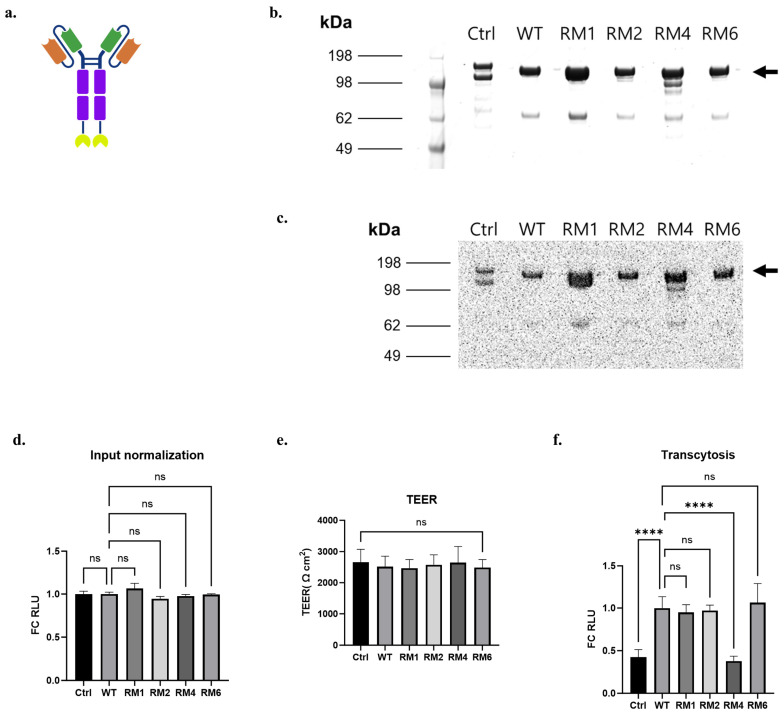
scFv-Fc-nLuc transcytosis across iPSC-BMEC-like cells for high-throughput screen variants. (**a**) Schematic of the scFv-Fc-nLuc construct. Orange: Variable heavy chain (VH), Green: Variable light chain (VL), Purple: Fc region, Yellow: nLuc. (**b**) Coomassie-stained non-reducing SDS-PAGE of purified, soluble scFv-Fc-nLuc. Arrow marks the size of full-length, dimeric scFv-Fc-nLuc. (**c**) Luminescence signal of each band of protein gel. (**d**) Luminescence signals (Relative Luminescence Units, RLU) of the scFv-Fc-nLuc fusion proteins applied to the apical chamber for the transcytosis assay expressed as fold change (FC) relative to the WT. (**e**) TEER of iPSC-BMEC-like cells used for the assay. (**f**) Luminescence signals of the basolateral chamber contents after four hours of transcytosis at 37 °C, expressed as FC relative to the WT. (**d**–**f**) were analyzed with one-way ANOVA, followed by Tukey’s multiple comparisons test. n = 6 transwells per sample, Ctrl is 4420-Fc negative control. **** *p* ≤ 0.0001, ns: not significant.

Given the ability of RM 1, 2, 4, and 6 to bind and internalize into iPSC-derived BMEC-like cells, we next quantified the functional transcytosis phenotypes of the 46.1 variants and compared them to the WT. For this assessment, we designed a construct where nLuc was genetically fused to the carboxy-terminus of the Fc region via an amino acid linker ((G_3_S)_2_) to create scFv-Fc-nLuc (Figure 4a). The use of nLuc allows for sensitive and quantitative assessment of transcytosis of small amounts of protein into the basolateral chamber. SDS-PAGE analysis followed by Coomassie staining validated production of the full-length scFv-Fc-nLuc constructs of the 4420 (negative control), WT, and the RM1, RM2, RM4, and RM6 variants at about 140 kDa, with some smaller breakdown products (Figure 4b). In-gel detection of luminescence signals generated by the nLuc fusion proteins indicated that the nLuc constructs were functional in the scFv-Fc-nLuc format, and each visible protein band possessed the nLuc portion of the fusion (Figure 4c). Thus, using the total protein concentration of the purified scFv-Fc-nLuc fusions, we dosed each protein at 153 nM to match the apparent dissociation constant (K_D_) of the WT 46.1-scFv-Fc binding to iPSC-BMECs [24]. We chose to dose at WT equilibrium binding conditions to allow variants having differences in binding, trafficking and release to translate into potential differences in transcytosis. Sampling of the scFv-Fc-nLuc proteins before dosing onto the apical chamber indicated that the amount of nLuc dosed to the iPSC-BMEC-coated transwells for each variant was indistinguishable (Figure 4d). In addition, the TEER values exceeded 2000 Ω cm^2^ for the assay and were indistinguishable across the negative control, WT, and the variants, ruling out differences in passive diffusion (Figure 4e). After four hours of transcytosis at 37 °C, we measured the accumulated scFv-Fc-nLuc in the basolateral chamber. As expected, WT 46.1-Fc-nLuc demonstrated about 2-fold higher transport across the iPSC-BMEC-like cell monolayer compared to the 4420-Fc-nLuc negative control. Variants RM1, RM2, and RM6 exhibited transcytosis levels indistinguishable from the WT. RM4 exhibited significantly lower transcytosis compared to the WT and was at the levels of the 4420-Fc-nLuc negative control (Figure 4f). Taken together, while the antibody variants increased in frequency during the phenotypic transcytosis screen, they did not perform better than the WT as soluble proteins.

### 3.3. CDR Histidine Mutation

**Figure 5:**
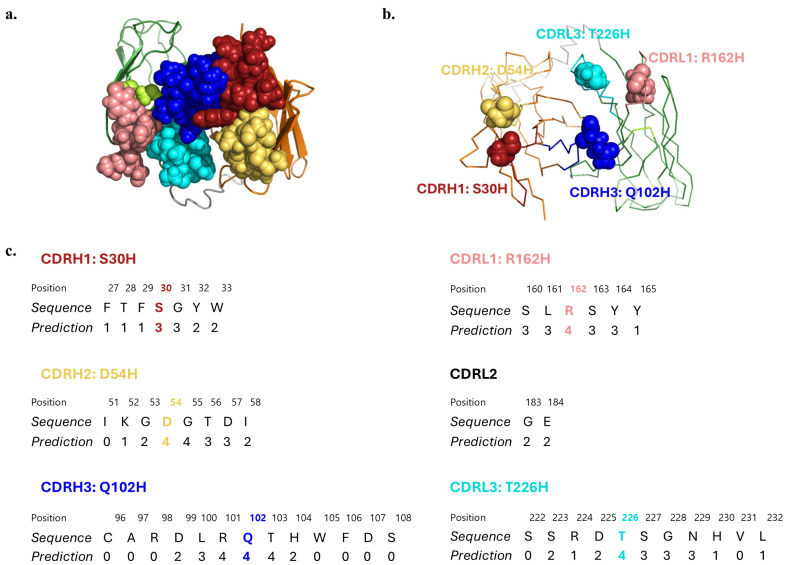
Homology model of scFv 46.1. (**a**) Cartoon diagram of the top view of the scFv with CDRs depicted in space filling representation. (**b**) Ribbon diagram with the residue of each CDR chosen for histidine substitution indicated in space filling representation. (**c**) Solvent accessibility prediction values of the residues in each CDR. Values range from 0 (buried residue) to 9 (highly exposed residue). Mutated residues are marked with colors. Red: CDRH1, yellow: CDRH2, blue: CDRH3, pink: CDRL1, lime: CDRL2, cyan: CDRL3, gray: (Gly3Ser)4 linker, orange: heavy chain, green: light chain. 2.5 Å starting template resolution; C-score = 0.85, where the C-score represents the confidence of each model, in the range of [−5, 2], where a higher C-score signifies a model with a higher confidence [56,57,58].

As a second strategy to potentially increase transcytosis across the iPSC-derived BBB model, we hypothesized that histidine point mutations of select CDR residues could increase the transcytosis efficiency of the antibody, driven by the fact that CDRs play a central role in antigen binding [39,41,59,60], mutations in the surface residues are less likely to disrupt protein stability [61,62,63], and histidines can confer pH sensitivity [44,47,48,64,65,66]. Since the molecular structure of scFv 46.1 has not been experimentally determined, we generated a homology model using the I-TASSER server [56,57,58] to provide a prediction of the tertiary structure and the solvent exposure values for the CDR residues (Figure 5). Choosing residues with the highest predicted exposure values, and thus more likely to interact with the RMT antigen, we generated the following variants: S30H (CDRH1), D54H (CDRH2), Q102H (CDRH3), R162H (CDRL1), and T226H (CDRL3) (Figure 5c).

### 3.4. Evaluation of Binding, Internalization, and Transcytosis of Targeted Histidine CDR Variants in iPSC-Derived BBB Model

**Figure 6:**
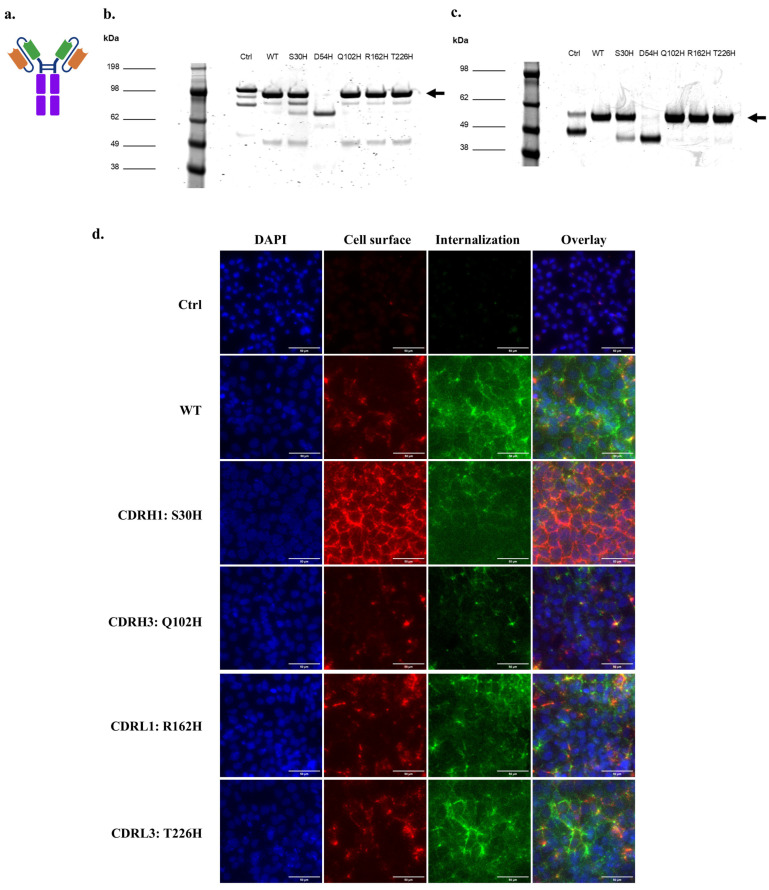
scFv-Fc binding and internalization into iPSC-BMECs for targeted histidine CDR variants. (**a**) Schematic of the scFv-Fc structure. Orange: Variable heavy chain (VH), Green: Variable light chain (VL), Purple: Fc region. (**b**) Coomassie-stained non-reducing SDS-PAGE of purified, soluble scFv-Fc. Arrow marks the size of full-length, dimeric scFv-Fc. (**c**) Coomassie-stained reducing SDS-PAGE of purified, soluble scFv-Fc. Arrow marks the size of one arm of the scFv-Fc. (**d**) Cell surface association and internalization of soluble scFv-Fc protein into iPSC-BMECs after 30 min incubation at 37 °C. Scale bars are 20 µm. Ctrl is 4420-Fc negative control.

Next, we produced soluble histidine variant proteins in scFv-Fc formats (Figure 6a). SDS-PAGE analysis followed by Coomassie staining indicated that full-size scFv-Fc could be produced for the WT 46.1 and the variants S30H, Q102H, R162H, and T226H with a dominant full-length dimeric band at the expected size of about 100 kDa (Figure 6b,c). The negative control 4420-Fc had some breakdown products at a lower molecular weight of about 80 kDa as noted previously (Figure 6b), and the variant D54H-Fc did not produce full length protein and was not evaluated further (Figure 6b). To confirm the capabilities of the variants to bind and internalize into iPSC-derived BMEC-like cells, scFv-Fcs were pulsed onto iPSC-derived BMEC-like cells and allowed to bind and traffic for 30 min each. All variants tested maintained their capability to internalize into the iPSC-derived BMEC-like cells and traffic to the cell junctions, although qualitatively, S30H demonstrated higher total cell surface labeling, and Q102H had lower overall cellular association (Figure 6d).

**Figure 7:**
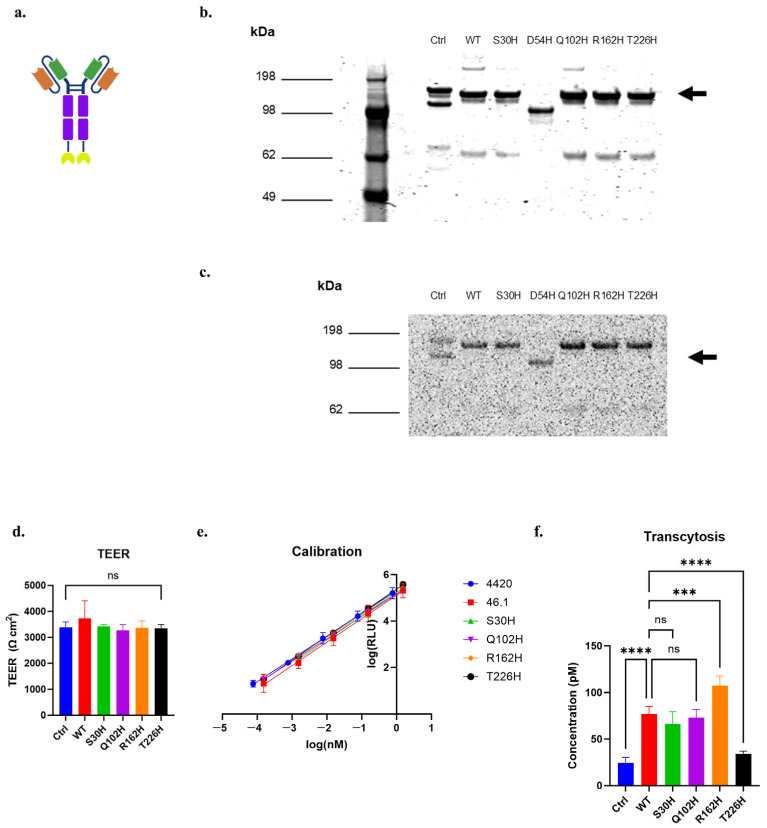
scFv-Fc-nLuc transcytosis across iPSC-BMEC-like cells for targeted histidine CDR variants. (**a**) Schematic of the scFv-Fc-nLuc construct. Orange: Variable heavy chain (VH), Green: Variable light chain (VL), Purple: Fc region, Yellow: nLuc. (**b**) Coomassie-stained non-reducing SDS-PAGE of purified, soluble scFv-Fc-nLuc. Arrow marks the size of full-length, dimeric scFv-Fc-nLuc. (**c**) Luminescence signal of each band of the non-reducing protein gel. Arrow marks the size of full-length, dimeric scFv-Fc-nLuc. (**d**) TEER of iPSC-BMEC-like cells used for the assay. (**e**) Calibration curves of luminescence units (RLU) versus protein concentration for each variant protein. (**f**) Protein concentration of the basolateral chamber contents after four hours of transcytosis at 37 °C, (**d**,**f**) were analyzed with one-way ANOVA, followed by Tukey’s multiple comparisons test. n = 4–6 transwells per sample. Ctrl is 4420-Fc negative control. *** *p* ≤ 0.001, **** *p* ≤ 0.0001, ns: not significant.

Given the ability of S30H, Q102H, R162H, and T226H to bind and internalize into iPSC-derived BMEC-like cells, we next quantified the functional transcytosis phenotypes of the variants and compared them to the WT using the nLuc-based transcytosis assays on iPSC-derived BMEC-like cells. For this, we again produced scFv-Fc-nLuc soluble proteins of the WT, 4420 (negative control), and the histidine variants (Figure 7a). Non-reducing SDS-PAGE analysis followed by Coomassie staining indicated the successful production of the full-length scFv-Fc-nLuc constructs of the negative control, WT, and the S30H, Q102H, R162H, and T226H variants at about 140 kDa, with some smaller breakdown products (Figure 7b). In-gel detection of luminescence signals generated by the nLuc fusion proteins indicated that the nLuc constructs were functional in the scFv-Fc-nLuc formats, and each major protein band possessed the nLuc portion of the fusion (Figure 7c). For these assays, we used iPSC-BMEC-like cells with TEER values exceeding 3000 Ω cm^2^ (Figure 7d). We dosed each protein at 153 nM, and after four hours of transcytosis, we measured the accumulated scFv-Fc-nLuc in the basolateral chamber and calculated their concentrations based on the luminescence–concentration calibration curves generated for each protein variant (Figure 7e). The transcytosis of R162H was 1.4-fold increased than the WT, while T226H was 0.4-fold reduced, and the other variants were indistinguishable from WT (Figure 7f). Taken together, these results suggest that the targeted histidine mutation of the 46.1 CDRs, followed by the transcytosis assessment on iPSC-BMECs, identified variants that translate into functional changes in in vitro transcytosis.

## 4. Discussion

Advances in antibody engineering strategies have accelerated the development of therapeutics for neurological diseases, but effective in vitro strategies for screening and evaluating antibody variants for altered BBB transport properties are limited. In this investigation, we introduce two main strategies in an attempt to improve in vitro BBB-traversing capacity of an antibody: directed evolution and targeted CDR mutagenesis. Subsequently, we report a quantitative in vitro transcytosis assay with scFv-Fc-nLuc fusion protein constructs and iPSC-derived BMEC-like cells to assess relative transcytosis efficiencies of individual variants to identify which variants lead to increased transcytosis across the BBB model. In this way, we identified clone R162H, which exhibited a modest improvement in in vitro transcytosis.

While we did identify variants that enriched in the transcytosis screen outputs and had different BBB transport characteristics compared with the WT (e.g., RM4) from the phage screening strategy, we were not able to identify variants with improved in vitro transcytosis properties. One significant challenge in these screens was identifying an appropriate selection pressure to distinguish the WT from improved variants. Key considerations included dosage, incubation times, and library composition. First, we employed iPSC-derived BMECs with TEER values exceeding 1000 Ω cm^2^, which effectively blocked paracellular diffusion of phage. In the absence of paracellular diffusion, there is a roughly 9-orders of magnitude decrease in the number of phage output compared to the input (10^11^ input phage vs. 100’s of output phage CFU). Thus, even for the WT BBB transcytosing antibody, only a very small percentage of phage-displayed antibody of the WT scFv can undergo the complete process of internalization, trafficking, and transcytosis process across the iPSC-BMEC model [24]. Because of this, one needs to properly oversample the input library, which we attempted to do by a 10^6^-fold excess of phage library added to the apical chamber. Even so, a variant that appears with low frequency in the initial library could still be missed. Second, we employed shorter transcytosis times because they resulted in lower phage accumulation in the basolateral chamber, indicating an effective application of screening pressure for variants that might pass through the BBB model more quickly or in higher frequency. Given that the basolateral output for the 60 min screen was about 7 CFU per transwell, even shorter transcytosis times would not be realistic. We chose to pool the outputs of the 90 min screen, balancing between variant diversity and screen stringency to enrich for the functional variants. Combined with a second round of 90 min screening, we were able to see clear enrichment of certain variants, whereas the basal WT percentages increased very little. Yet, when expressed as soluble proteins, the transcytosis properties were not improved. Thus, as a different approach to increase the yield and fitness of improved variants, a library that encompasses more multi-mutation variants and takes bigger steps in the phenotypic transcytosis landscape could lead to identification of more promising lead candidates [67,68]. Of course, this approach would be limited by the size of phage display libraries that could be practically synthesized along with potential disruptions to protein function with higher mutational pressure [67,69].

Interestingly, the most enriched variants had mutations in the antibody framework regions including the DE loop (Table 1, Supplementary Figure S3) [70]. While antigen binding is often associated with CDRs, regions outside the CDRs can affect factors such as protein stability, folding, and CDR conformations, and thereby influence engagement with receptors [39,60,70,71,72,73,74]. Residues in the framework regions in proximity to the CDRs can, on occasion, bind antigens, and those distant from the CDRs can alter the conformations of the CDRs and the Fv domains and thus significantly affect binding specificity [39,60,74]. The variants explored having framework mutations did not significantly improve BBB transcytosis in this study, although the most enriched variant RM6 contained a mutation N199D in the DE loop, the fourth loop adjacent to CDR1 and CDR2, which can also be involved in ligand interactions [70]. Despite the enrichment in framework variants, we speculate that the CDRs may impact transcytosis more substantially since CDRs typically constitute the core of the antigen-binding region [39,41,60]. Therefore, future efforts using the screening platform presented here could benefit from CDR-targeted mutagenic libraries.

Along these lines, we turned to a second strategy of introducing point histidine mutations at the most solvent-exposed residue of individual CDRs, given that histidines can confer pH sensitivity [44,47,48,64,65,66]. Indeed, increased pH-dependent trafficking across the BBB has been demonstrated for several TfR-targeting antibodies, due to enhanced dissociation at endosomal pH (pH 5.5) versus physiological pH (pH 7.4) [44,47,48]. The variant which had mutation in the CDRL1 loop, R162H, exhibited modestly enhanced in vitro transcytosis compared to the WT antibody (Figure 7f). The mutation of the positively charged arginine into a histidine residue changes the mutated residue’s charge from +1 to 0 at pH 7.4 but retains the same positive charge as the WT residue at endosomal pH (pH 5.5). While purely speculative, it is possible that the R162H variant is influencing the interaction of the antibody with the cognate RMT receptor and impacting the transcytosis machinery. Moreover, point mutation of a single residue is unlikely to cause dramatic effects on pH-induced effects on receptor association or dissociation of the antibody from its receptor. In fact, many engineered pH-sensitive antibody variants require three or more histidine mutations [44,47,65,66]. Thus, future work may benefit from histidine saturation mutagenesis either on the CDRL1 for which the point mutation resulted in enhanced in vitro transcytosis, or the CDRL3 which had a high mutational frequency in the Round II output of the 90 min random mutagenesis screen (Figure S3b). Of note, the enzymatic in vitro transcytosis assay using the iPSC-derived BBB model was able to detect and discriminate transcytosis of protein variants accumulating at picomolar concentrations in the basolateral chamber. In addition, a critical component of this BBB model platform is that TEER values were exceeding 1000 Ω cm^2^, a tightness which has been shown to be important for limiting the passive diffusion of IgG [75]. Thus, if a BBB model that does not have high TEER is used, passive diffusion of the antibody would mask the true antibody transcytosis which occurs at a slower rate.

Finally, a limitation of in vitro assessments in BBB models is that it is unclear if increased in vitro transcytosis of a variant like R162H would translate to increased brain uptake after systemic administration. In previous studies, when used in a pharmacodynamic assay, the WT antibody led to a pharmacologically relevant hypothermic response in mice upon neurotensin conjugation [25]. Thus, it will be important in the future to evaluate improved variants identified in the in vitro screens in pharmacodynamic assays to fully assess their brain uptake potential and whether they would warrant further development.

## 5. Conclusions

In summary, the present study introduces strategies to engineer and evaluate antibody variants with relevant functional phenotypes. We expect that this phenotype-driven early discovery approach could be extendable to other antibody-BBB RMT systems that lack complete mechanistic understanding.

## Figures and Tables

**Table 1:** Frequency of occurrence of lead candidate clones in the input library and the outputs of each screen. N.D.: not detected.

Clone	Mutations	Input Library	60-Min	90-Min		120-Min
				Round I	Round II	
WT		11.73%	26.32%	13.91%	14.13%	19.92%
RM1	N84S I207T	<0.01%	0.00%	0.98%	5.43%	1.32%
RM2	K172E	0.02%	5.26%	0.49%	4.35%	N.D.
RM3	S118P A143V Y169H M181V I208T	<0.01%	0.00%	N.D.	3.26%	N.D.
RM4	A88T P177L	<0.01%	5.26%	0.00%	1.09%	N.D.
RM5	L138P A217T	<0.01%	5.26%	1.96%	2.17%	0.44%
RM6	S186G N199D	<0.01%	0.00%	1.47%	6.52%	1.75%

## Data Availability

The original contributions presented in this study are included in the article/Supplementary Materials. Further inquiries can be directed to the corresponding author.

## Supplementary Materials

The following supporting information can be downloaded at: https://www.mdpi.com/article/10.3390/antib14040102/s1, Figure S1: Frequency of mutations in the random mutagenic 46.1 library at each amino acid position. Figure S2: Characterization of iPSC-derived BMEC-like cells. Figure S3: Full screen clonal output.
