# Supplementary material for: Strategies to Screen and Evaluate Brain Targeting Antibodies Using an iPSC-Derived Blood–Brain Barrier Model
Source: Antibodies (Basel). 2025 Nov 26;14(4):102. doi: 10.3390/antib14040102 (PMC12729367; doi:10.3390/antib14040102)
Supplement: Supplementary file 1 [file antibodies-14-00102-s001.zip › antibodies-3963477-supplementary.pdf]

Supplementary Figures

a.

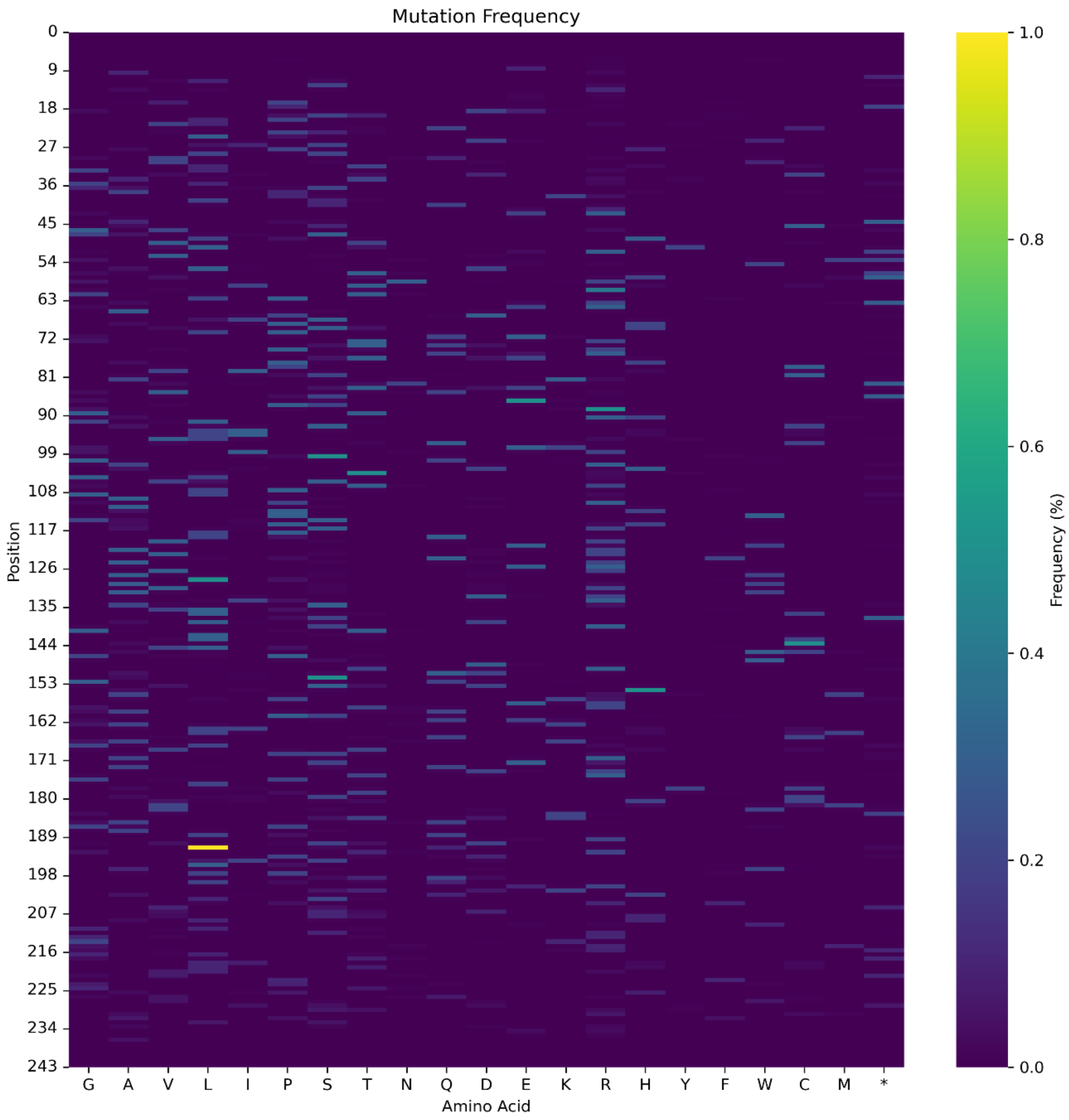

b.

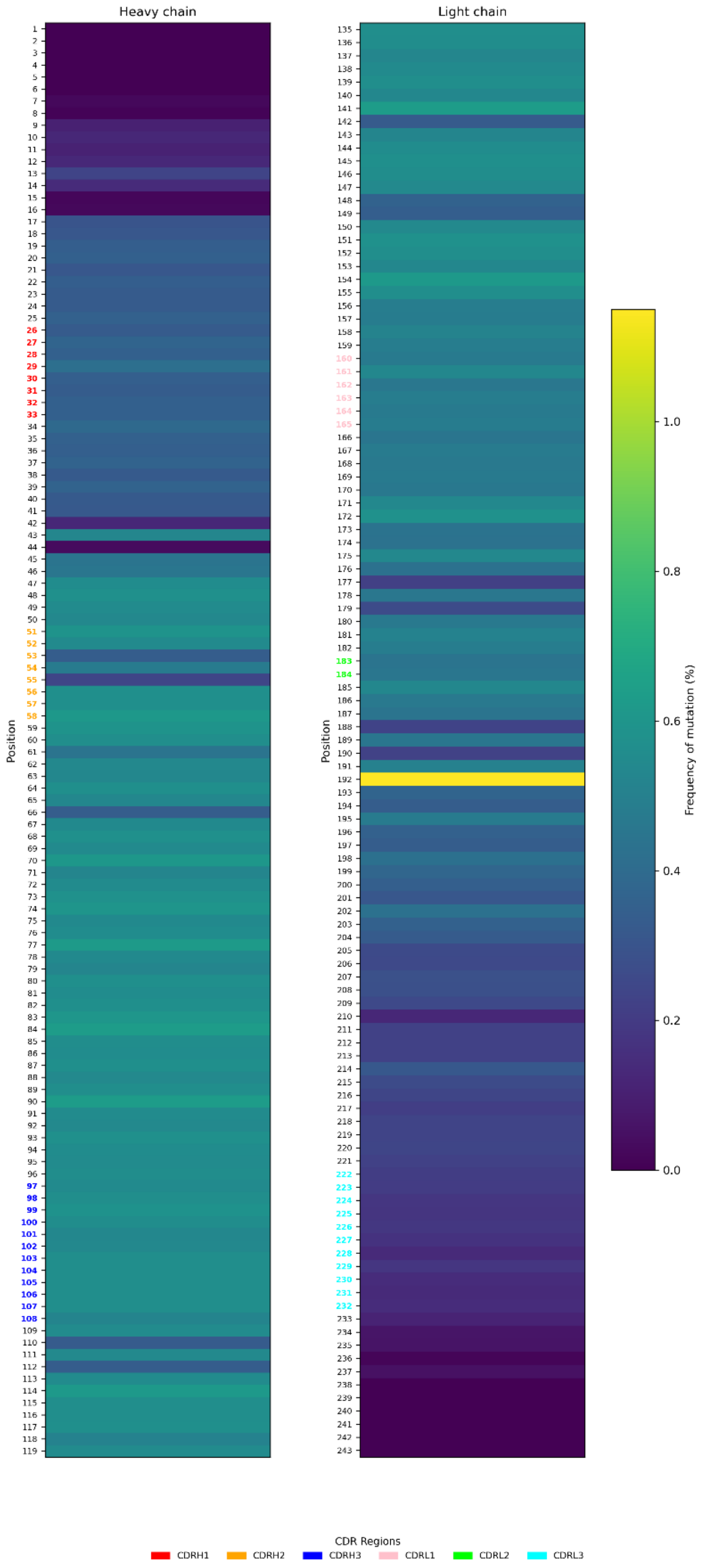

Supplementary Figure S1: **Frequency of mutations in the random mutagenic 46.1 library at each amino acid position. a.** Heatmap of the frequency of mutation at each amino acid position into individual amino acid residues. **b.** Heatmap of the total frequency of mutation at each position, summing across all amino acid substitutions, for the heavy and light chains. CDR residues are color-coded into CDRH1: Red, CDRH2: Yellow, CDRH3: Blue, CDRL1: Pink, CDRL2: Lime, CDRL3: Cyan.

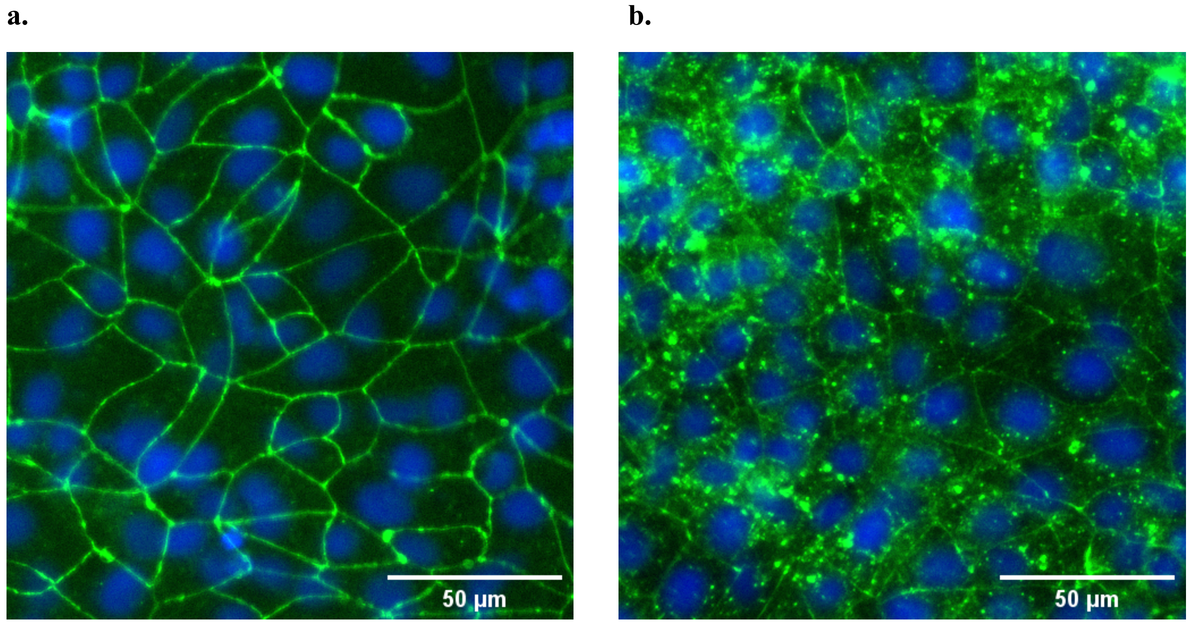

Supplementary Figure S2: Characterization of iPSC-derived BMEC-like cells. Immunocytochemistry of iPSC-BMEC-like cells probing for the expression of tight junction proteins **a.** Occuldin and **b.** Claudin-5. Scale bars are 50 µm.

a.

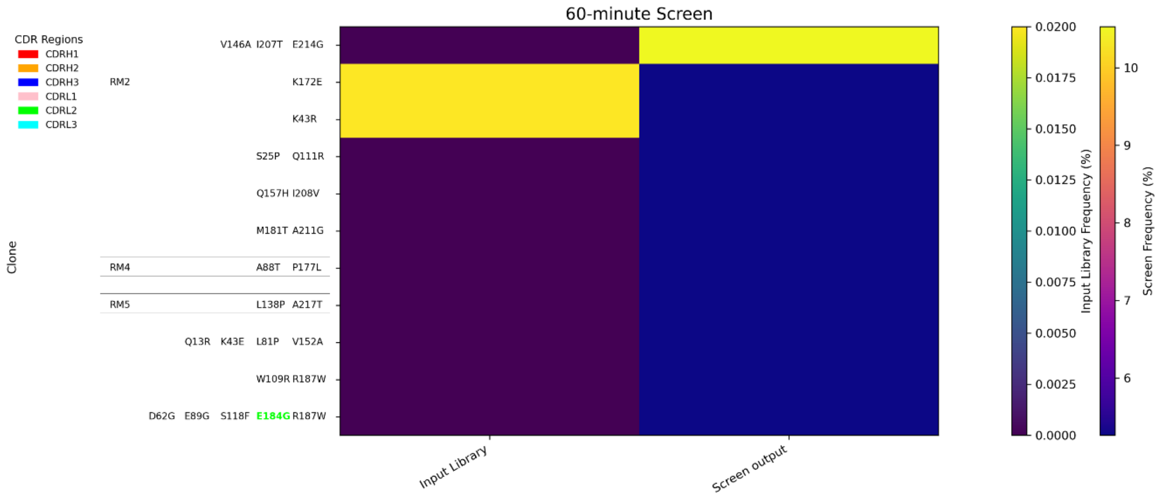
60-minute Screen
CDR Regions
CDRH1
CDRH2
CDRH3
CDRL1
CDRL2
CDRL3
Clone
V146A I207T E214G
RM2 K172E
K43R
S25P Q111R
Q157H I208V
M181T A211G
RM4 A88T P177L
RM5 L138P A217T
Q13R K43E L81P V152A
W109R R187W
D62G E89G S118F E184G R187W
Input Library
Screen output
Input Library Frequency (%)
Screen Frequency (%)

b.

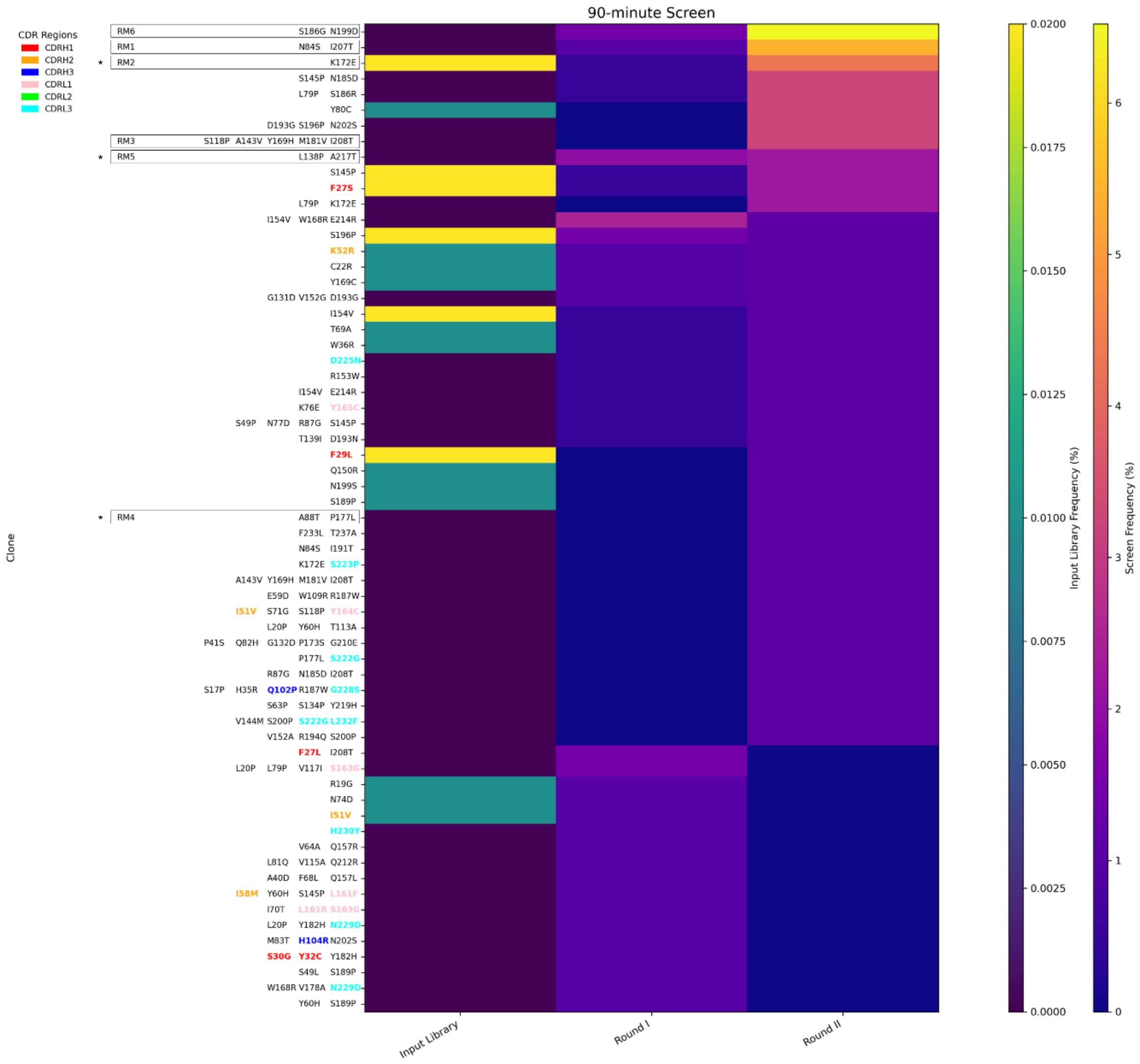
90-minute Screen
CDR Regions
CDRH1
CDRH2
CDRH3
CDRL1
CDRL2
CDRL3
Clone
RM6 S186G N199D
RM1 N84S I207T
RM2 K172E
S145P N185D
L79P S186R
Y80C
D193G S196P N202S
RM3 S118P A143V Y169H M181V I208T
RM5 L138P A217T
S145P
F27S
L79P K172E
I154V W168R E214R
S196P
K52R
C22R
Y169C
G131D V152G D193G
I154V
T69A
W36R
D225N
R153W
I154V E214R
K76E Y165C
S49P N77D R87G S145P
T139I D193N
F29L
Q150R
N199S
S189P
RM4 A88T P177L
F233L T237A
N84S I191T
K172E S223P
A143V Y169H M181V I208T
E59D W109R R187W
I51V S71G S118P Y164C
L20P Y60H T113A
P41S Q82H G132D P173S G210E
P177L S222G
R87G N185D I208T
S17P H35R Q102P R187W G228S
S63P S134P Y219H
V144M S200P S222G L232F
V152A R194Q S200P
F27L I208T
L20P L79P V117I S163G
R19G
N74D
I51V
H230Y
V64A Q157R
L81Q V115A Q212R
A40D F68L Q157L
I58M Y60H S145P L161F
I70T L161R S163G
L20P Y182H N229D
M83T H104R N202S
S30G Y32C Y182H
S49L S189P
W168R V178A N229D
Y60H S189P
Input Library
Round I
Round II
Input Library Frequency (%)
Screen Frequency (%)

c.

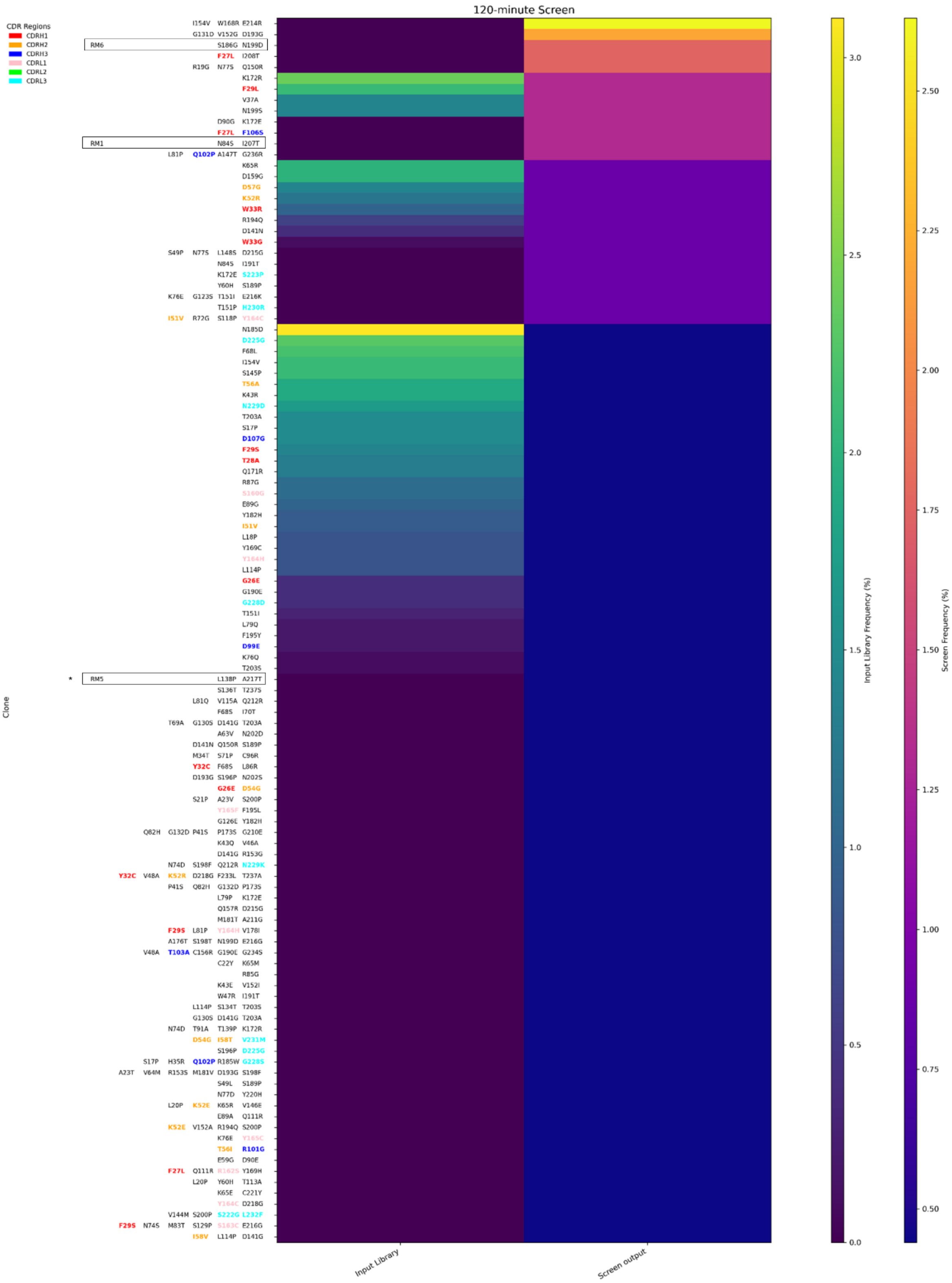

Supplementary Figure S3: Full screen clonal output. Heatmap of the frequency of occurrence of each clone present in the output of the **a.** 60-minute, **b.** 90-minute, and **c.** 120-minute screens. Mutations into stop codons were omitted. Selected lead candidates are marked with boxes. Asterisks indicate the clones that appeared in the 60-minute screen output. CDRs are color-coded. CDRH1: Red, CDRH2: Yellow, CDRH3: Blue, CDRL1: Pink, CDRL2: Lime, CDRL3: Cyan.
